# Global elimination of HBV: Is it really achievable?

**DOI:** 10.1111/jvh.13955

**Published:** 2024-05-26

**Authors:** Erwan Vo‐Quang, Maud Lemoine

**Affiliations:** ^1^ Disease Control & Elimination Theme Medical Research Council Unit The Gambia at London School of Hygiene & Tropical Medicine Banjul The Gambia; ^2^ Team “Viruses, Hepatology, Cancer”, Institut Mondor de Recherche Biomédicale, INSERM U955 Université Paris‐Est Créteil France; ^3^ Department of Metabolism, Digestion and Reproduction, Division of Digestive Diseases, St Mary's Hospital Imperial College London London UK

**Keywords:** hepatitis B virus, hepatitis C virus

## Abstract

Hepatitis B virus (HBV) infection is a major cause of premature death worldwide. In 2016, the World Health Organization (WHO) called for HBV elimination and set up very ambitious elimination targets. The development of effective vaccines, accurate diagnostic tools and safe antiviral drugs make HBV elimination a realistic goal. However, the most constrained‐resource regions, which bear the highest burden of HBV, are facing major challenges in implementing strategies to reduce HBV incidence and mortality. Developing simplified approaches adapted to resource‐limited settings and scaling up interventions for the prevention and control of HBV globally are urgently needed. Whether HBV elimination will be achieved in an equitable manner and in a reasonable timeframe remains highly uncertain.

**Abbreviations**
ALT: alanine transaminase
CHB: chronic HBV infection
DNA: deoxyribonucleic acid
GBD: Global Burden of Disease
HBcrAg: hepatitis B Core‐Related Antigen
HBsAg: hepatitis B surface antigen
HBV: hepatitis B virus
HCC: hepatocellular carcinoma
HEPB3: 3 doses of hepatitis B vaccine
HepB‐BD: hepatitis B birth dose
HIV: human immunodeficiency virus
LMICs: low‐ and middle‐income countries
PMTCT: prevention of mother‐to‐child transmission
RDT: rapid diagnostic test
SDI: Socio‐Demographic Index
WHA: The World Health Assembly
WHO: The World Health Organization

## 1 INTRODUCTION

In 2013, 1.5 million deaths due to viral hepatitis were reported, and viral hepatitis emerged as the seventh‐leading cause of mortality globally, surpassing HIV infection, tuberculosis, and malaria.^1^ Consequently, for the first time, the World Health Assembly (WHA) recognized viral hepatitis as a threat to global health and included the fight against viral hepatitis among the 2030 sustainable development goals. The World Health Organization (WHO) developed a viral hepatitis elimination strategy with the ambition to decrease new viral hepatitis cases and related mortality by 90% and 65%, respectively, by 2030.^2^

Because hepatitis B virus (HBV) infection affects many more people (296 million) than hepatitis C virus (HCV) (58 million) globally,^3^ and in the absence of curative drugs for HBV, eliminating HBV appears as a more challenging goal compared to HCV elimination. The most recent estimates on HBV incidence and HBV‐related mortality are far behind the 2030 elimination targets, and so far, only 40 countries, including 37 countries in Europe and Americas, have already reached the HBV elimination objectives.^4^ Nevertheless, HBV elimination is not an unrealistic aim, as a wide range of low‐cost interventions, are available and have been proven effective in reducing HBV incidence and related mortality. One of the remaining questions related to global HBV elimination is when and how HBV will be eliminated without exacerbating global inequalities. While more than 40% of countries in the American and European regions have already reached the 2030 HBV elimination targets, less than 5% in other regions have met these targets.^4^ Access to and coverage of interventions for the prevention and the control of HBV infection widely differ between countries and between geographical areas and communities within the same country, posing a risk to equitable elimination.

## 2 GLOBAL HEPATITIS B DYNAMICS

### 2.1 HBV elimination criteria

The global strategy endorsed by the WHA in 2016 to achieve HBV elimination aims for a 90% reduction in new infections (incidence) and a 65% reduction in deaths (mortality) by 2030.^2^ To reach these ‘impact targets,’ the WHO emphasizes a strategy focused on four main interventions: Hepatitis B vaccination, HBV Prevention of Mother‐to‐Child Transmission (PMTCT), testing services and treatment. The indicators employed for monitoring and evaluating these interventions, such as 3 doses of hepatitis B vaccine (HEPB3) coverage, hepatitis B birth dose (HepB‐BD) coverage, the percentage of HBV‐infected diagnosed and the percentage of diagnosed with HBV on treatment, are established at target rate of 90%, with the exception of the last indicator, for which the target is set at 80%.^5^ In 2021, the WHO altered the terminology for HBV elimination, transitioning from a relative reduction definition to absolute prevalence, incidence, and mortality targets, established as the WHO Interim Guidance provisional target for 2030.^6^ As HBV transmission predominantly occurs in young children, achieving a 90% reduction in new infections by 2030 is tantamount to obtaining a reduction in the prevalence of hepatitis B surface antigen (HBsAg) to below 0.1% in children under 5 years, which is used as a proxy for HBV incidence. Additionally, mortality has been redefined as annual mortality (incidence), which should be less than 4/100,000 people/year. Furthermore, the PMTCT programmatic target has been expanded, adding three programmatic targets to the existing ones. These include antenatal care (at least one visit), HBsAg antenatal testing, and antiviral prophylaxis for eligible HBsAg‐positive pregnant women at risk of HBV MTCT. The goal is to achieve and maintain a 90% target for at least 2 years.^6^ These targets help the WHO regions and countries in assessing whether they are on track to eliminate HBV. Targets for 2020 (WHO‐GHSS for 2020) and 2030 (WHO Interim Guidance provisional target for 2030) are summarized in Table 1.

**TABLE 1:** Targets for achieving HBV elimination by 2020 and 2030.

Target for achieving HBV elimination		By 2020		By 2030	
Impact targets	Relative targets	30% reduction in incidence	10% reduction in mortality	95% reduction in incidence	65% reduction in mortality
	Absolute targets	≤1% HBsAg prevalence in ≤5 years old	NA	≤0.1% HBsAg prevalence in ≤5 years old ≤2% MTCT rate (for countries with targeted HepB‐BD)	≤4/100000
Programmatic targets	HepB3 vaccine coverage	≥90%		≥90%	
	HepB‐BD coverage	≥50%		≥90%	
	Coverage of maternal antenatal HBsAg testing (for countries with targeted HepB‐BD)	NA		≥90%	
	Coverage with prophylaxis for those eligible (for countries with targeted HepB‐BD)	NA		≥90%	
	People with HBV diagnosed	≥30%		≥90%	
	People diagnosed with HBV and eligible for treatment are treated	5 million (10.0%) of those eligible for treatment		≥80%	

*Note*: Global health sector strategy on viral hepatitis (GHSS) 2016–2021 defined relative targets.^2^ WHO. Interim guidance for country validation of viral hepatitis elimination, 2021 defined absolute targets.^6^

Abbreviations: HBsAg, hepatitis B surface antigen; HBV, hepatitis B virus; HepB3, three doses of hepatitis B vaccine (infant vaccination); HepB‐BD, hepatitis B birth dose vaccine; MTCT, mother‐to‐child transmission; NA, not applicable.

### 2.2 Global burden of hepatitis B and its disparities

In 2019, the WHO estimated that 296 million (3.8%) people have chronic HBV infection (CHB) worldwide and approximately 1,500,000 new infections and 820,000 deaths were attributed to HBV.^3^ Worldwide HBV accounts for 42% of cirrhosis cases^7^ and is a major cause of hepatocellular carcinoma (HCC).^8^ Age‐standardized HBsAg seroprevalence is the highest in the African Region (7.5%), followed by the Western Pacific region (5.9%) (Table 2).^3^

**TABLE 2:** Estimation of HBsAg prevalence in the general population and among children under 5 years, derived from reports by Global Burden of Disease, The World Health Organization and Polaris.

	Global Burden of Disease	The World Health Organization	Polaris
Year of publication	2019	2020	2022
Prevalence of HBsAg in the general population			
Global	4.1%	3.8%	3.2%
African region	6.5%	7.5%	5.4%
Region of the Americas	1.2%	0.5%	0.6%
South‐East Asia region	3.1%	3.0%	3.8%
European region	1.1%	1.5%	1.8%
Eastern Mediterranean region	3.1%	2.5%	2.9%
Western Pacific region	7.1%	5.9%	7.1%
Hepatitis B in children younger than 5 years			
Global	1.0%	0.9%	0.7%
African region	2.7%	2.5%	1.7%
Region of the Americas	<0.1%	<0.1%	<0.1%
South‐East Asia region	0.5%	0.4%	0.6%
European region	0.1%	0.3%	<0.1%
Eastern Mediterranean region	0.8%	0.8%	0.4%
Western Pacific region	0.5%	0.3%	0.3%

*Note*: Data from Global Burden of Disease 2019,^4^ WHO report 2019,^3^ Polaris 2022.^9^

Globally, the prevalence of HBsAg in children under 5 years is 1%, with an estimated number of infections at 6.4 million.^3^, ^4^ All WHO regions have a prevalence of HBsAg in children under 5 years less than 1%, except the African region, where it is 2.5% (Table 2).^3^ The 2019 Global Burden of Disease (GBD) data highlighted a significant reduction (of 77% since 1990) in global HBsAg prevalence among infants under 5 years.^4^ However, this reduction was mostly observed in Western^10^ and Asian countries.^4^

**FIGURE 1:**
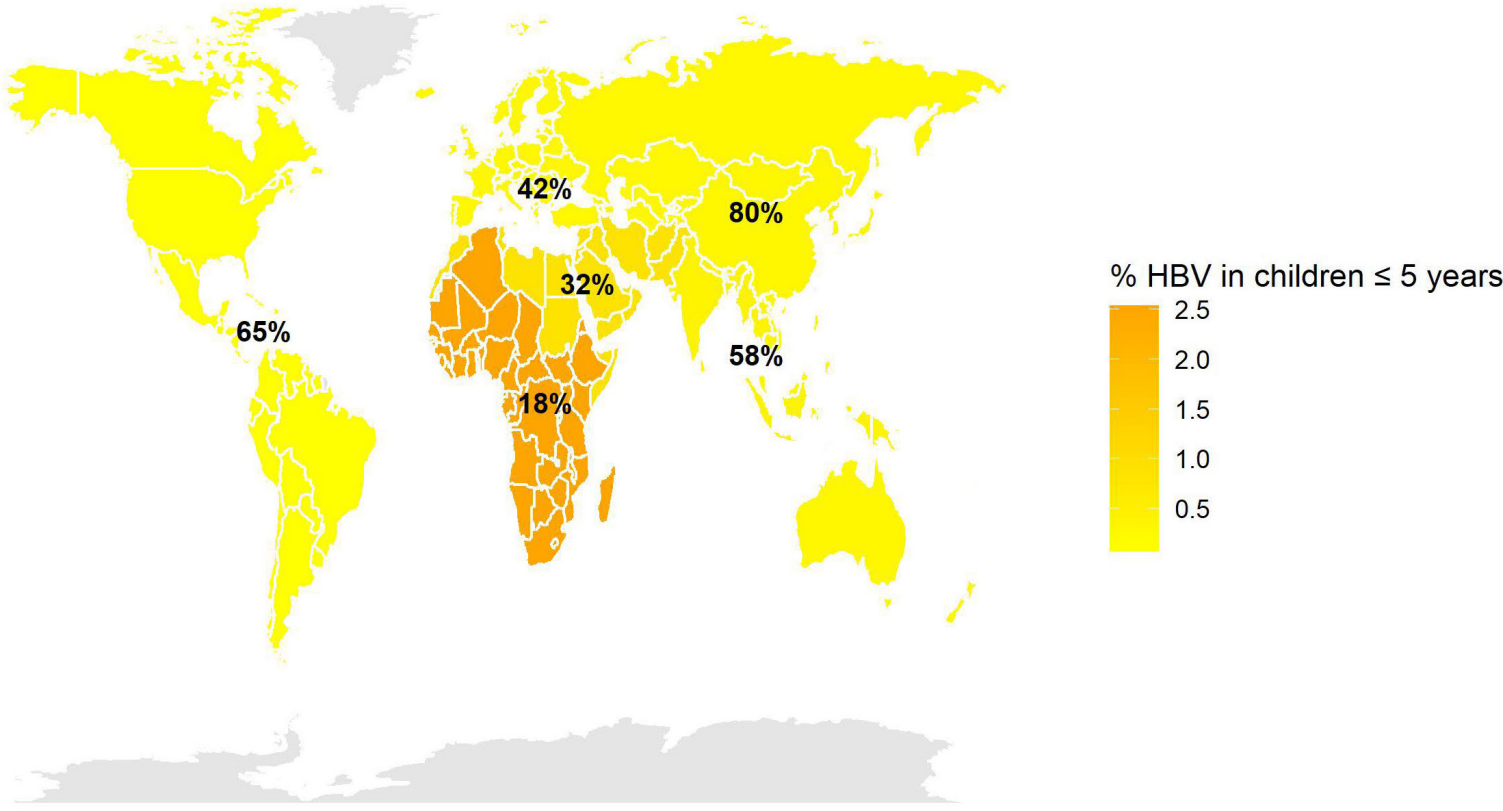
HBsAg prevalence in children below 5 years and HepB‐BD vaccine coverage by the WHO region. The prevalence of HBsAg in children under 5 years is sourced from the WHO report 2019.^3^ The figure has been supplemented with 2022 immunization data from the WHO by region.^11^

As suggested by Figure 1, global HBsAg prevalence in infants under 5 years by the WHO regions is correlated with HepB‐BD vaccine coverage. The 2016 Polaris Observatory data estimated an annual incidence of 1.8 million in children, highlighting that HBV incidence worldwide occurs mostly through MTCT or during early childhood.^3^, ^12^ Eighty percent of new infections were observed in 16 countries, with four countries (Nigeria, India, Indonesia, and the Democratic Republic of the Congo) accounting for almost 57% of new infections.^12^

In addition to geographical disparities, the burden of CHB also widely varies among specific populations. While the HBsAg prevalence in pregnant women is usually comparable to that in the general population in the same geographic area,^13^ other vulnerable populations, such as sex workers, people who inject drugs,^14^, ^15^ homeless individuals,^16^ people living with HIV, patients on haemodialysis,^17^ and prisoners^18^ are at higher risk of HBV infection.

Finally, more than two third of people with CHB reside in low‐ and middle‐income countries (LMICs).^3^ In addition, the burden of CHB is geographically distributed in countries with low Socio‐Demographic Index (SDI). HBsAg seroprevalence in children under 5 years, and mortality rates in countries with low SDI are much higher compared to countries with high SDI.^4^

### 2.3 Towards global but unequal progress to HBV elimination

The Global Burden of Disease (GBD) Study 2019 HBsAg prevalence (4.1%), reflected a 31% decrease from the 1990 prevalence.^4^ However, the absolute number of HBV‐related deaths in 2019 increased by 6% and 3% from 1990 and 2015, respectively.^4^ The decrease in new HBV cases and HBV‐related mortality was mainly observed in high‐income countries, leaving LMICs behind the HBV elimination objectives.

According to recent reports, the WHO‐GHSS for 2020 new cases impact target, aiming for a 30% reduction in new HBV infections between 2015 and 2019, was achieved in only 15/194 countries (8%).^4^ However, 147/194 (76%) countries met the proxy target based on prevalence in children under 5 years (<1%) by 2020. This target was accomplished by all countries in Europe and Americas but only by 15 out of 47 (32%) countries in Africa. Regarding the mortality objectives, which aimed for a 10% reduction in deaths between 2015 and 2019, only four countries (Namibia, Montenegro, Ireland, and Dominica) achieved this target.^4^

Estimations of programmatic targets offer an overview of intervention coverage. In 2022, 14% of CHB patients were diagnosed, and 8% of eligible population were treated.^9^ The highest diagnosis coverage did not exceed 26% in WHO regions and was mainly observed in high‐income countries, while it was estimated at 4% in African and 3% in South‐East Asian regions.^9^ A similar unequal distribution was observed regarding the proportion of treated CHB people, with 1% and less than 1% receiving antiviral in Africa and Southeast Asia, respectively.^9^ Regarding the coverage of infant vaccination with three recommended doses at 4, 8 and 12 weeks age (HepB3) and the coverage of timely HepB‐BD, the WHO‐GHSS 2020 targets, were not achieved in 2022, with global estimates at 84% and 45%, respectively^11^ and estimates even lower in the WHO Africa (72% for HepB3 coverage and 18% of HepB‐BD).^11^ Estimations of the programmatic targets are summarized in Table 3.

**TABLE 3:** The level of programmatic targets globally and across WHO Africa and Europe regions in 2022.

Interventions	Estimates		
	Globally	Africa	Europe
HepB3 vaccine	84%	72%	91%
HepB‐BD	45%	18%	42%
Prophylaxis for pregnant mothers eligible	3%	<1%	5%
People with HBV diagnosed	14%	4%	20%
People diagnosed with HBV and eligible for treatment are treated	8%	1%	10%

*Note*: Data from WHO/UNICEF 2022 estimates^11^ and Polaris 2022.^9^

Abbreviations: HBV, hepatitis B virus; HepB3, three doses of hepatitis B vaccine (infant vaccination); HepB‐BD, hepatitis B birth dose vaccine.

**FIGURE 2:**
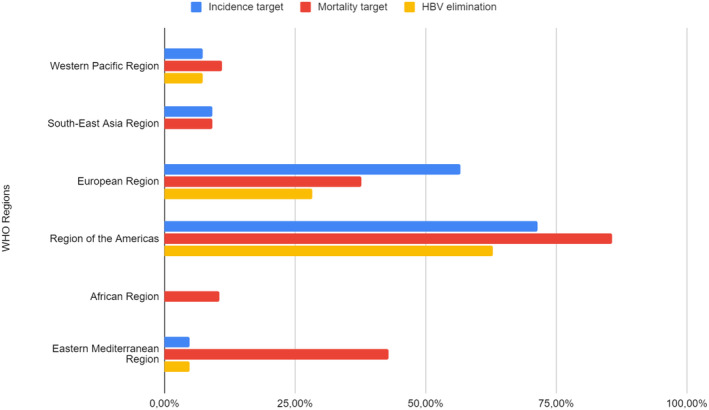
Proportion of countries by WHO region that met or exceeded in 2019 WHO Interim Guidance provisional targets by 2030. Adapted from Global Burden of Disease 2019.^4^

The WHO Interim Guidance provisional targets by 2030 were met for several countries, mostly in high‐income countries. Of the 194 countries included in GBD Study 2019, 59 (30%) met or exceeded the proxy target of no more than 0.1% HBsAg seroprevalence in infants and children under 5 years, and 68 (35%) had already achieved an all‐age mortality rate of less than or equal to 4/100,000 people/year.^4^ Nevertheless, worldwide, only 40 (21%) countries, including 15 in the European Region and 22 in the Regions of the Americas, met both WHO Interim Guidance provisional targets in 2019 (Figure 2). In the WHO Africa Region, none of the 47 countries and only 5 out of 47 met or exceeded the WHO Interim Guidance provisional targets for 2030 on prevalence and on mortality, respectively.^4^

## 3 SCALING UP INTERVENTIONS TO ACCELERATE HBV ELIMINATION

A large number of inexpensive interventions including HBV vaccination and antiviral therapy are now widely available in most countries. Unfortunately, these interventions have been inadequately implemented putting at high risk the HBV global elimination objectives.

### 3.1 HBV PMTCT

HBV MTCT has become the main route of HBV transmission globally, as suggested by a modelling study, which highlighted that the success of infant vaccination starting at 6 weeks will lead to a shift to a higher contribution of MTCT in new infections in all regions (50% by 2030).^19^ Besides the high rate of HBV transmission attributable to MTCT, it is urgent to prevent MTCT for the following reasons. First, it is well‐established that the risk of developing CHB when the virus is acquired at birth is high (>95%).^20^ Moreover, subjects infected from mother‐to‐child have a higher risk of liver complications compared to subjects contaminated later during childhood. In The Gambia, West Africa, a longitudinal analysis of children with CHB found that the risk of significant liver fibrosis was 5.0 times higher in those born to HBsAg‐positive mothers than in those born to HBsAg‐negative mothers.^21^ Routine infant HepB immunization, including HepB‐BD vaccination, is a highly effective public health strategy that reduces HBsAg prevalence, HBV‐related liver disease, HCC, and premature mortality.^22^, ^23^, ^24^

#### 3.1.1 HepB‐BD vaccine

Since 2009 to prevent early transmission of HBV, the WHO recommends the universal administration of a timely HepB‐BD vaccine, within the first 24 h of birth in all newborns. Scaling up infant vaccination, including HepB‐BD, has led to a major reduction in HBsAg prevalence in children worldwide, from 4.7% in the pre‐vaccination era to 1.3% in 2015.^5^ However, with an estimation at 45%, the coverage of HepB‐BD globally remains very low.^11^ The global adoption of the HepB‐BD policy has not been yet materialized, with only 58% of countries (114 out of 195) introducing it into their routine immunization schedules in 2020.^25^ The WHO Africa region, where only 14 out of 47 countries have adopted a HepB‐BD policy,^26^ has the lowest coverage most recently estimated at 18%.^11^

Several barriers from low awareness to need of cold chain can explain this low coverage. Our recent study which analysed about 71,000 births in The Gambia, found that being born on Fridays and Saturdays, outside medical facilities, during the rainy season or during the COVID‐19 pandemic, were strongly associated with delayed HepB‐BD in babies born between 2015 and 2021 (unpublished data). Second, the decision by GAVI to pause their support for HepB‐BD provision during the COVID‐19 pandemic had major negative impact in Africa. Third, low awareness in healthcare workers and limited logistical support, including vaccine supply and distribution.^27^ Fourth, poor awareness among pregnant mothers is another key obstacle to improve HepB‐BD coverage.^28^ Finally, in the 1143 regions across 37 countries in sub‐Saharan Africa and South Asia, 28% of births occurred at home.^29^ The lack of outreach programs to reach babies born outside health facilities is another major barrier to the timely delivery of the HepB‐BD vaccine.^27^

Two studies from Ivory Coast and Cameroon highlighted a reduction of HBsAg prevalence among the HepB‐BD cohort.^30^, ^31^ But, considering the major difficulties of implementing HepB‐BD in Africa and following the absence of clear benefit of HepB‐BD for prevention of early transmission of HBV in babies born to HBsAg‐negative women in Africa,^32^ a targeted approach, that is, administrating HepB‐BD only in babies born to HBsAg‐positive women may improve HepB‐BD coverage in LMICs; but this remains uncertain. Moreover, an economic analysis in São Tomé and Príncipe, central Africa showed that a universal HepB‐BD strategy without maternal screening was cost‐saving compared with the existing selective HepB‐BD strategy, largely due to the cost of maternal screening.^33^

#### 3.1.2 Antiviral prophylaxis in pregnant women

Despite the administration of infant HepB‐BD immediately after birth, residual HBV MTCT has been reported in babies born to high viraemic women (i.e with a viral load ≥200,000 IU/mL or tested positive HBeAg).^31^ As the result, it is widely recommended to treat high viraemic women with TDF during pregnancy.^34^ Sadly, the most recent estimates on global uptake of peripartum antiviral prophylaxis in women at risk of MTCT, is only around 3%.^9^

Yet, the efficacy and safety of antiviral therapy for HBV PMTCT has been well documented, mainly from Asian trials.^35^ Data on the effectiveness of maternal antiviral prophylaxis from LMICs, especially from Africa, is scarce. A study from Cambodia reported the safety and efficacy of TDF during pregnancy in mother at risk of HBV MTCT^36^ and Thompson et al. also found in a small group of highly viremic women that TDF is effective to prevent HBV MTCT.^37^

The identification of mothers at risk of MTCT based on HBV viral load is a major challenge in LMICs. While the GeneXpert^®^ system is now widely used by the tuberculosis and HIV country programmes and COVID‐19 pandemic has led to the implementation of additional GeneXpert machines in most LMICs, its access in rural areas is limited and it requires regular maintenance and stable electricity supply. Mothers at risk of HBV MTCT can also be identified using HBeAg serology. However, serology is also difficult to perform in routine in most LMICs and the poor accuracy of HBeAg rapid diagnostic tests (RDTs) cannot allow its use to select pregnant women for antiviral prohylaxis.^38^

The development of innovative RDTs that can assess HBV viral load, could lead to a significant change in the management of HBsAg‐positive pregnant women. A novel RDT for Hepatitis B Core‐Related Antigen (HBcrAg) may improve the identification of highly‐viremic mothers.^39^, ^40^ In settings where measurement of HBV DNA or HBeAg serology is not accessible, a prophylaxis‐all strategy, consisting in treating all HBsAg pregnant women irrespective of their HBV viral load level, has been increasingly considered as a pragmatic alternative approach to eliminate HBV MTCT in LMICs.^41^ But the feasibility and effectiveness and cost effectiveness of such a strategy needs to be assessed in real‐life settings. A new policy, called triple elimination of MTCT, has been suggested by the WHO, aiming to integrate HBV infection within the HIV and syphilis services and control MTCT of HIV, Syphilis and HBV.^42^ Such a strategy has shown effectiveness in Mozambique,^43^ but additional data is needed, especially in Africa.

### 3.2 Case finding, diagnosis and treatment

Case finding of HBV could be easily improved by offering a systematic screening of HBsAg using RDTs at least once during adulthood. Indeed, compared to HIV, people tested negative for HBsAg are likely to remain negative during the rest of their life since acute HBV infection during adulthood is associated with a very high rate of spontaneous HBV clearance.

One of the main concerns regarding the management of CHB currently relies on the complexity of CHB staging, which requires several assessments based on measurement of at least HBV DNA, ALT level and liver fibrosis. These tests are not widely accessible especially in LMICs. The use of simple and inexpensive point‐of‐care tests may overcome this barrier. The GeneXpert^®^ system may overcome this difficulty,^44^, ^45^ and may even shorten the time to initiate HBV treatment.^46^ Point‐of‐care ALT has been developed but need further validation in real life.^47^

Detection of early cirrhosis and HCC surveillance are also essential to reduce HBV‐related morbidity and mortality but are facing many barriers in LMICs. Globally, one in 10 of the millions CHB people (26 million) might be in urgent need of treatment because of cirrhosis.^48^ However, in most HBV endemic countries, cirrhosis and HCC are usually diagnosed at late stage.^49^ With excellent performance for the detection of cirrhosis, transient elastography using FibroScan^®^ should help for the detection of cirrhotic cases.^50^ However, access to Fibroscan^®^ is not accessible in remote and decentralized areas. Until recently, the WHO recommended the implementation of aspartate aminotransferase‐to‐platelet ratio index (APRI) to detect liver fibrosis. However, the majority (81%–89%) of patients with advanced fibrosis or cirrhosis were missed by these scores.^51^ An individual patient data meta‐analysis from African studies of CHB patients proposed a lower threshold (<0.36) to rule out cirrhosis with a sensitivity and specificity of 80.6% and 64.3%.^52^

As a result, complexity of CHB staging and inaccessibility of these tests lead to the absence of liver assessment in resource‐limited countries. The vast majority of CHB patients in need of treatment cannot be identified and do not have access to treatment. Progress has been made to reduce cost of HBsAg RDTs and antiviral therapy (tenofovir disoproxil fumarate [TDF] costs less 30 USD per year) but ensuring access to treatment is crucial to achieve the global viral hepatitis elimination targets set by the WHO.

Moreover, half of the patients in need of treatment are not detected by the current 2016 WHO treatment criteria^53^ which has been revised in the WHO 2024 guidelines.

Following studies suggesting that patients in the ‘grey zone’ may be at risk of HCC^54^, ^55^ and would benefit from treatment,^56^ expanding antiviral treatment criteria is currently considered by the WHO and most international liver societies. Another hypothetical strategy, inspired by the fight against HIV, is a ‘treat‐all’ approach, where antiviral treatment could be offered to all individuals tested positive for HBsAg, irrespective of their ALT levels, viral loads, or degree of liver fibrosis. This ‘treat‐all’ approach cannot be widely recommended due to lack of evidence. Moreover, whether such an approach will be cost‐effective in LMICs remains debated.

Finally, integration of CHB treatment within other services, such as the HIV or tuberculosis services, could improve the cascade of care and clinical outcomes, but this requires further evaluation.^57^

### 3.3 Children and adolescents with CHB: a neglected population

Knowledge on prevention, treatment, and management of children and adolescents living with CHB needs to be improved.^58^ Compared to adult population, children, and adolescents with CHB have been long neglected or are systematically excluded from clinical trials. Yet, the number of children migrating to Europe and North America is increasing.^59^ In Spain, among 373 immigrant children and adolescents coming from Africa, and Latin America, HBsAg prevalence was 4.2%.^60^ As HBV infection during childhood leads to chronic HBV infection and cirrhosis, screening for HBV infection should be discussed systematically when children from endemic areas are attending hospitals in high‐income countries.

### 3.4 Strengthening data collection systems

There is a need to collect accurate data on the global burden of CHB. The WHO, GBD, and Polaris estimations employ diverse methodologies but exhibit consistency in estimating HBsAg prevalence among adults globally and across WHO regions. (Table 2). However, significant differences are observed in country‐level prevalence estimates among young children, especially in Africa.^61^ In addition, these estimates do not include patients with resolved HBsAg seroprevalence who remain at risk of developing liver cancer, especially in the case of cirrhosis,^62^ and occult HBV infection.^63^, ^64^ Finally, deaths caused by HBV in Africa account for 10% of total deaths worldwide,^3^ but estimates might be underestimated as deaths due to HBV liver cancer are not well reported in that region.^65^ Moreover, to estimate the effort of countries on HBV elimination, age‐stratified serosurveys need to be done to assess the prevalence of HBsAg.

A recent network, HEPSANET (Hepatitis B in Africa Collaborative Network), has been developed and aims to characterize the natural history of HBV infection in Africa.^66^ More than eight countries are collecting data. Risk factors for liver disease progression, HCC incidence and mortality will be assessed.

### 3.5 Funding and political support

Even if HepB‐BD vaccine, HBV RDTs, and TDF are affordable, they remain hardly available in most resource‐limited countries, especially in Africa, Unlike HIV, malaria, and TB, countries cannot apply for funding to support HBV infection in their own country. Recently, the Global Hepatitis Resource Mobilization Conference was held in May 2023, organized by The Hepatitis Fund and the Clinton Health Access Initiative, aiming to establish a new international funding mechanism.^67^ Even if a yearly course of TDF was set at $29, the target amount (US$150 million) is low and will not make HBV elimination feasible. It is well known now that individual interventions are cost‐effective in nearly all scenarios.^68^ Even if existing governmental and non‐governmental agencies such as like GAVI, Unitaid, Clinton Health Access Initiative will support HBV elimination objective, there is an urgent need to allocate additional funding for the elimination of HBV. A parallel with the HIV response can be made. Thanks to commitment from the global health sector, new interventions were rapidly developed and led to a significant decrease in morbidity and mortality.^69^

Despite commitment in 2021 from GAVI to assist resource limited‐countries to scale up of HepB‐BD vaccination, GAVI halted its plan and supported instead COVID‐19 vaccinations. Respondents from 19 (79%) of 21 countries in Africa reported the immediate availability of Gavi funds was of high importance.^70^ The recent decision of GAVI to finance the HepB‐BD vaccine will support the commitment of the Global Fund, UNICEF, WHO, and other agencies to the global strategy for the triple EMTCT of HIV, HBV, and syphilis by 2030.^42^

## 4 HEPATITIS B ELIMINATION PROJECTIONS IN THE 21ST CENTURY

A mathematical model found that lack of scaling‐up recommended interventions would lead to 63 million new cases of CHB and 17 million HBV‐related deaths between 2015 and 2030.^19^ In case of status quo, another model reported 47 million new cases of CHB, 7 million of new HCC of 7 million, and 18 million deaths attributable due HBV by 2050.^68^

Improving HBV PMTCT strategy that includes infant vaccination to 90%, HepB‐BD vaccination, and peripartum antiviral therapy to 80% will prevent a further 19.3 million new infections, with most of that incremental impact achieved by HepB‐BD vaccination alone (18.7 million new cases prevented).^19^ However, even with scaling up interventions, HBV incidence elimination will only be reached in half of the world's regions before 2060.^19^ Maintaining HepB‐BD vaccine could lead to elimination by 2030 in the Americas, but not before 2059 in Africa.^71^ Indeed, this is dramatic in West Africa, where HBV incidence elimination will only be reached in 2090.^19^

HBV mortality elimination can be achieved only if testing and treating interventions (peripartum antivirals and treatment) are added. 80% coverage of these interventions could reduce deaths by 65%, averting 7.3 million deaths, including 1.5 million cancer deaths, between 2015 and 2030, compared with the status quo.^19^ Most of the region worldwide will achieve that target after 2050.

Delaying the time when these interventions are achieved (from 2030 to 2050) has a major impact on cumulative incident chronic HBV infections (+10 million), hepatocellular carcinoma cases (+1 million) and HBV attributable deaths (+4 million).^68^ It would also have an impact on cumulative productivity losses, with an increase from 544 billion US dollars in 2030 to 691 billion US dollars in 2050.^68^

## 5 CONCLUSION

Major progress has been made to control the burden of HBV globally. Effective interventions are available, and HBV global elimination should theoretically be possible. However, major regional disparities in accessing interventions for the control and elimination of HBV, put at risk the elimination objectives in an equitable manner and in a reasonable timeframe. LMICS, especially the African countries, have been left behind and are facing the most difficult challenges to eliminate HBV. Combined efforts involving researchers, clinicians, policy makers and funders are urgently needed to see this HBV elimination aim becomes reality.

## CONFLICT OF INTEREST STATEMENT

ML received research funding and consultancy fees from Gilead Sciences and Abbott, USA. EV‐Q has no conflict of interest.
